# Geographic accessibility to public health facilities providing tuberculosis testing services at point-of-care in the upper east region, Ghana

**DOI:** 10.1186/s12889-019-7052-2

**Published:** 2019-06-10

**Authors:** Desmond Kuupiel, Kwame M. Adu, Felix Apiribu, Vitalis Bawontuo, Duncan A. Adogboba, Kwasi T. Ali, Tivani P. Mashamba-Thompson

**Affiliations:** 1 Department of Public Health Medicine, School of Nursing and Public Health, University of KwaZulu-Natal, Durban, South Africa; 2 Department of geography, University of Ghana, Legon, Ghana; 3 Department of Nursing, Kwame Nkrumah University of Science and Technology, Kumasi, Ghana; 4 Faculty of Health and Allied Sciences, Catholic University College of Ghana, Fiapre, Sunyani, Ghana; 5 Regional Health Directorate, Ghana Health Service, Upper East Region, Bolgatanga, Ghana; 6 Catholic Health Services, Goaso Diocese, Goaso, Brong Ahafo Region Ghana; 7 Research for Sustainable Development Consult, Sunyani, Ghana

**Keywords:** Geographic, Distribution, Accessibility, Health facilities, Tuberculosis, Point-of-care testing, Upper east region, Ghana

## Abstract

**Background:**

In Ghana, limited evidence exists about the geographical accessibility to health facilities providing tuberculosis (TB) diagnostic services to facilitate early diagnosis and treatment. Therefore, we aimed to assess the geographic accessibility to public health facilities providing TB testing services at point-of-care (POC) in the Upper East Region (UER), Ghana.

**Methods:**

We assembled detailed spatial data on all 10 health facilities providing TB testing services at POC, and landscape features influencing journeys. These data were used in a geospatial model to estimate actual distance and travel time from the residential areas of the population to health facilities providing TB testing services. Maps displaying the distance values were produced using ArcGIS Desktop v10.4. Spatial distribution of the health facilities was done using spatial autocorrelation (Global Moran’s Index) run in ArcMap 10.4.1. We also applied remote sensing through satellite imagery analysis to map out residential areas and identified locations for targeted improvement in the UER.

**Results:**

Of the 13 districts in the UER, 4 (31%) did not have any health facility providing TB testing services. In all, 10 public health facilities providing TB testing services at POC were available in the region representing an estimated population to health facility ratio of 125,000 people per facility. Majority (60%) of the health facilities providing TB testing services in the region were in districts with a total population greater than 100,000 people. Majority (62%) of the population resident in the region were located more than 10 km away from a health facility providing TB testing services. The mean distance ± standard deviation to the nearest public health facility providing TB testing services in UER was 33.2 km ± 13.5. Whilst the mean travel time using a motorized tricycle speed of 20 km/h to the nearest facility providing TB testing services in the UER was 99.6 min ± 41.6. The results of the satellite imagery analysis show that 51 additional health facilities providing TB testing services at POC are required to improve geographical accessibility. The results of the spatial autocorrelation analysis show that the spatial distribution of the health facilities was dispersed (z-score = − 2.3; *p* = 0.02).

**Conclusion:**

There is poor geographic accessibility to public health facilities providing TB testing services at POC in the UER of Ghana. Targeted improvement of rural PHC clinics in the UER to enable them provide TB testing services at POC is highly recommended.

**Electronic supplementary material:**

The online version of this article (10.1186/s12889-019-7052-2) contains supplementary material, which is available to authorized users.

## Background

Tuberculosis (TB) remains a global health threat and is one of the leading causes of death particularly human immunodeficiency virus (HIV) positive patients or among people living with acquired immunodeficiency syndrome [1, 2]. In 2017, the World Health Organization (WHO) global estimates show that, approximately 10 million people became ill with TB and more than 1.5 million died from the disease [1]. Over 94% of all TB deaths occurred in low- and middle-income countries (LMICs) including Ghana [1]. Ghana is classified as one of the countries with the highest double burden of TB and HIV, with a combined TB/HIV mortality rate of 18 per 100,000 populations translating to about 10,440 deaths per annum [3]. Early diagnosis and treatment of TB have been proven to be of greater benefits reducing morbidity and mortality as well as catastrophic cost related to TB treatment [2, 4].

The Government of Ghana (GOG) in her quest to reduce the impact of TB on the population over the years have made efforts to expand TB diagnostic services in the country through procurement of GeneXpert MTB/RIF test for some rural health facilities and retooling of laboratories across the country’s hospitals. Despites the continuous effort been made by the GOG and international organization, in 2017, out of the 14,550 TB case notifications received in Ghana, 6% of the patients were tested with rapid diagnostics at the time of diagnosis [5]. An average of 986 multi drug-resistant TB (MDR-TB) cases were reported in Ghana in the same period [5]. It also estimated that just about a quarter of MDR-TB cases are been detected yearly in Ghana, of which just close to half get cured [6, 7].

To reduce the burden of TB morbidity and mortality, more patients with TB need to be screened, diagnosed, and treated or prevented from developing TB illness in the first place [2, 3]. Prevention of TB cases is particularly critical in rural and resource-limited settings, where access to point-of-care (POC) testing and diagnosis of patients with tuberculosis can be very much challenging. Most rural primary health care (PHC) facilities commonly lack laboratory infrastructure and the required human resource capacity to diagnose TB. One of the most effective ways to improve early diagnosis and treatment of patients with TB is to empower rural and resource-limited PHC clinics to be able to carry out POC testing for suspected TB patients. Our earlier survey of PHC clinics in the Upper East Region (UER), Ghana aimed at evaluating the accessibility of POC diagnostic services demonstrated poor availability of TB testing [8]. In view of this, we aimed to assess the geographic accessibility to public health facilities providing TB testing services at POC in the UER, Ghana. To the best of our knowledge, no such study has been conducted before in the region and, we anticipate that the results will be useful for planning to reduce the catastrophic cost associated with TB diagnosis and treatment as well as towards achieving the End TB Strategy by 2035 [9].

## Methods

### Overview

**Fig. 1:**
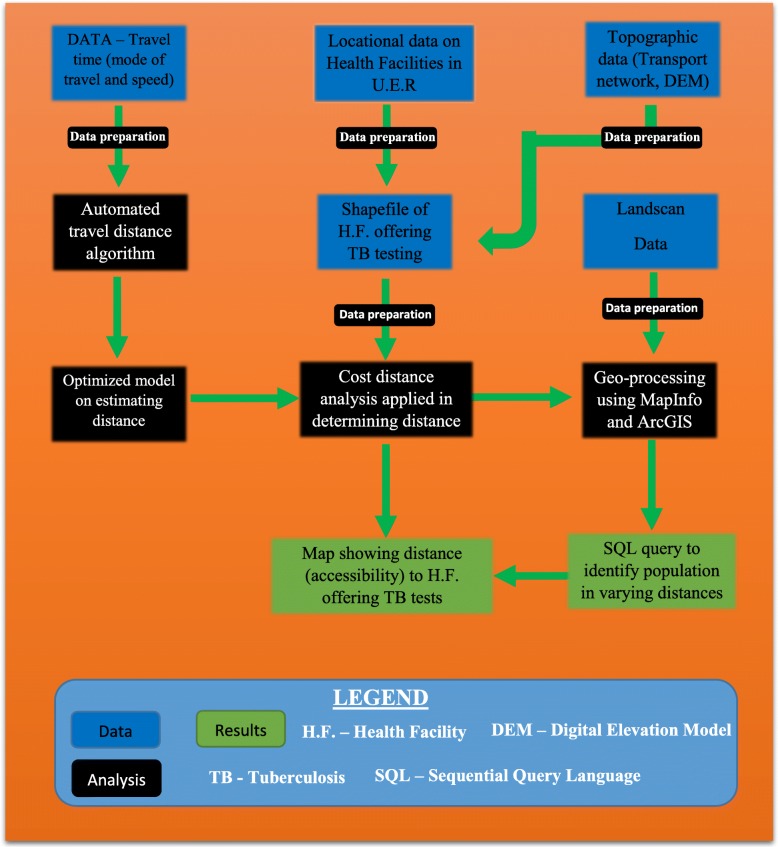
Process flow diagram for the procedures undertaken to arrive at the results

Figure 1 shows schematically the various data and modelling components used to achieve the objective of this study. The methodological aim of this study was to model geographical access of patients to health facilities providing TB testing services in the UER, Ghana. The region with 13 administrative districts is in the north-eastern corner of Ghana, bordered by Burkina Faso to the north, Togo and Upper West Region to the east, and Northern Region to the south. The region had 1,244,983 people in 2018 and is considered largely (79%) rural and scattered in dispersed settlements [10]. The main source of income for most of the population is farming.

The study spatially referenced or obtained locational data of all health facilities providing TB testing services via a prior survey aimed to assess the accessibility of POC diagnostic tests in the region [8], and the use of global positioning system (GPS). Thereafter, we applied the world geodetic system (WGS) Zone 30 North coordinate system to all spatial data in order to allow for results of spatial processes in a preferred unit of ‘meters’. Topographic data included roads, rivers and the digital elevation model (DEM). A previous survey in the region preceding this present study showed that motorized tricycle popularly known as “Moto king” was the most utilized public transport option [8]. We estimated travel time via road and paths/tracks based on the “Moto King” transport system. This involved recalibrating travel time per pixel (10 m by 10 m grid) for both roads and paths. This ultimately helped to estimate travel time from settlement areas of the population to health facilities offering TB testing for all the districts in the region.

### Geo-database of TB testing health facilities

Spatial and attribute (A-spatial) data on TB testing health facilities was collected and prepared by assigning one coordinate system to all the data to allow for synchronization. We then loaded the data in google earth to determine and confirm their location via landmarks and other reconciliatory features. The data was then imported into ArcGIS in the point shapefile format. The spatial data obtained primarily covered the ‘what’ and the ‘where’ that is, the clinic or hospital name and location (coordinates). Besides the name, other attribute data included the district where the facility is located (district name), the type of health clinic (health center, hospital, and others), whether they provided TB testing services and the commonly used public transport option. A cross-section survey prior to this study helped eliminate public health facilities that do not provide TB testing services from the analysis [8]. These were filtered out using the select by attribute function and eventually clipped.

### Topographic data

This data covered digitised and georeferenced road networks and paths well as features like rivers and lakes. The DEM of the region was also obtained to help identify natural barriers such hills and valleys as well as undulating land that would inform the decision on estimating travel time. All data was obtained from Adu Manu Kwame (AMK) Consult and juxtaposed with data obtained from the University of Ghana Remote Sensing and Geographic Information Systems laboratory to validate accuracy. The DEM was then reclassified into highlands (more than 200 m high) and flatlands (between 119 and 200 m high). This was determined by the DEM data which showed that the highest point in the region was about 470 m whiles the lowest point identified as 119 m.

### Model for estimating travel time

This was accomplished using a sequence of algorithms with the final output incorporating cost distance tool in ArcGIS 10.4. Cost distance calculates the shortest time to a source based on a cost dataset. To realize this, a cost surface algorithm was designed with these parameters: a grid cell of size 10 m was assigned to the spatial features and values were then assigned to the predetermined grids. Roads were assigned low values because traveling on roads is faster than travel via paths and travel over impediments such as water bodies and rocky areas takes a much longer time and hence assigned higher values. Since cost distance requires the cost surface dataset and the source, the raster dataset served as the cost surface dataset and health facilities providing TB testing served as the source for calculating the cost distance. The output is a map showing shortest travel time (cell by cell) from any point in the map to a health facility providing TB testing services in the region. Algorithms which allowed for carrying out conversion of data from vector to raster, map algebra (cost surface models) and cost distance was developed using Python 2.7. Designing the algorithm helped to avoid repeating the various processes for the varying districts via the ArcMap toolbox. Based on prior information on the most commonly used public transportation option in the UER, the travel speed of a motorised tricycle was pecked at 20 km/h. This served as a guide for determining travel time in the region. A detailed description of the model is presented in Additional file 1.

### Determining distance for population

The Oakwood Ridge National Laboratory Landscan data set serves as a standardized and globally accepted population distribution data [11]. This data was mapped in MapInfo and an SQL query was employed in clipping out the population distribution within all the available travelling distances from health facilities offering TB testing.

### Outcome measures

The primary outcome of this study was geographical accessibility to health facilities offering TB testing in UER, Ghana. Geographical accessibility was measured as: 0-10 km = good geographical access; and > 10 km = poor geographical access. Considering ≤10 km as good geographical accessibility be arbitrary. However, evidence shows that access to healthcare beyond 10 km is associated with higher risks of adverse outcomes [12]. Also, categorising geographical accessibility using travel time would have been useful but, travel time depends on the mode of transportation option and the route hence our choice to base our categorisation of geographical accessibility on distance. The secondary outcome of this study was to identify locations for targeted improvement of TB testing services in the UER utilizing geographical models and the application of remote sensing through satellite imagery analysis. Remote sensing also known as earth observation, refers to acquiring information about objects located on the earth’s surface without being in direct contact with the said object. The application of remote sensing through satellite imagery afforded us the opportunity to observe locations that had settlements and help us properly cite proposed health clinics without necessarily having to visit the region.

### Statistical analysis

Spatial autocorrelation tool or Global Moran’s I [13] was run in ArcMap 10.4.1 to determine the spatial distribution of the health facilities providing TB diagnostic services in the UER. The Global Moran’s I statistic was run with the region as the input feature class and the count of health facilities providing TB testing services in each district as the input field to determine the spatial distribution of the health facilities. Z-scores and *p*-values were generated and reported. Mean and standard deviation (SD) for distance and travel time were also reported.

## Results

### Distribution of the health facilities providing TB testing services in UER

In all, 10 public health facilities providing TB testing services at POC existed in the region This represented an estimated population to health facility ratio of 125,000 people per facility in the UER. Of the 10 facilities, 9 (90%) were owned by the Ghana Health Services, and the remaining one (10%) owned by the Presbyterian church, a member of the Christian Health Association of Ghana. Majority (60%) of the health facilities providing TB testing services in the region were in districts with a total population greater than 100,000 people. Two (20%) of the 10 facilities were in the Bawku West district, and one (10%) each in the remaining 8 (69%) districts and municipalities in the region. Four districts (Builsa South, Nabdam, Pusiga, and Binduri) did not have any health facility providing TB testing services located in the districts. Table 1 shows the population distribution per public health facility providing TB testing services in the UER based on the sum of health facilities providing the service in each district.

**Table 1:** Distribution of health facilities providing TB testing services per population in the UER

District	Name of facility (*N* = 10)	Population (*N* = 1,244,983)	Population/facility
Builsa North	Sandema hospital	67,190	67,190
Kassena Nankana West	Paga health center	84,585	84,585
Kassena Nankana Municipal	War Memorial hospital	130,593	130,593
Bolgatanga Municipal	Bolgatanga Regional hospital	156,678	156,678
Talensi	Tongo hospital	97,010	97,010
Bongo	Bongo hospital	100,741	100,741
Bawku West	Zeibilla hospital	105,970	105,980
	Binaba health center	5970	5960
Garu Tempane	Garu health center	154,214	154,214
Bawku Municipal	Bawku Presbyterian hospital	116,912	116,912
Builsa South	Not available	43,534	–
Nabdam	Not available	39,798	–
Binduri	Not available	73,382	–
Pusiga	Not available	68,406	–

### Spatial distribution of health facilities offering TB testing services in upper east region

**Fig. 2:**
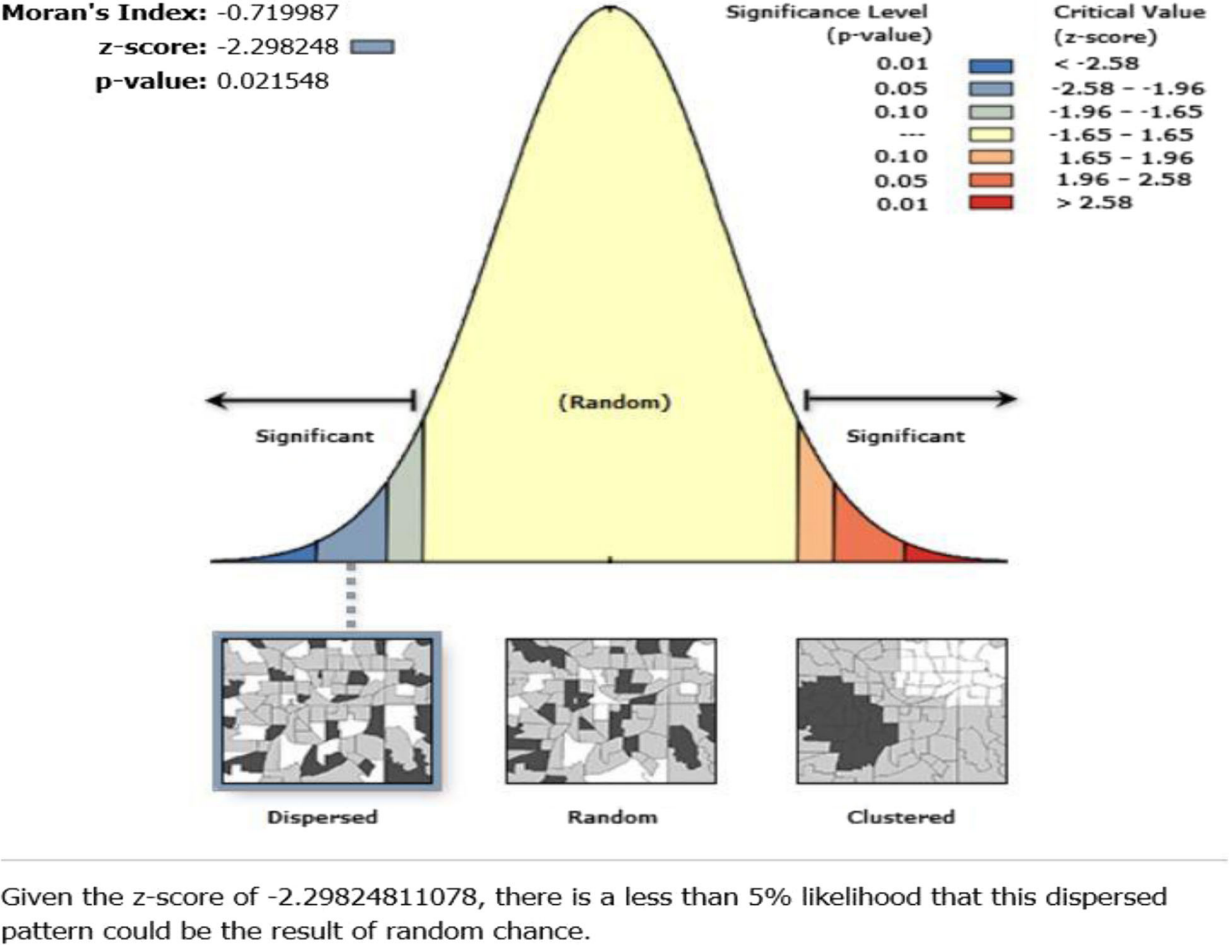
Spatial distribution of health facilities offering TB testing in the UER

The spatial autocorrelation analysis was carried out to determine how spatially distributed the health facilities providing TB testing services are, in the UER. The result shows that the health facilities were dispersed (z-score = − 2.3; *p* = 0.02), as illustrated in Fig. 2.

### Geographic accessibility to TB testing services in the upper east region

**Fig. 3:**
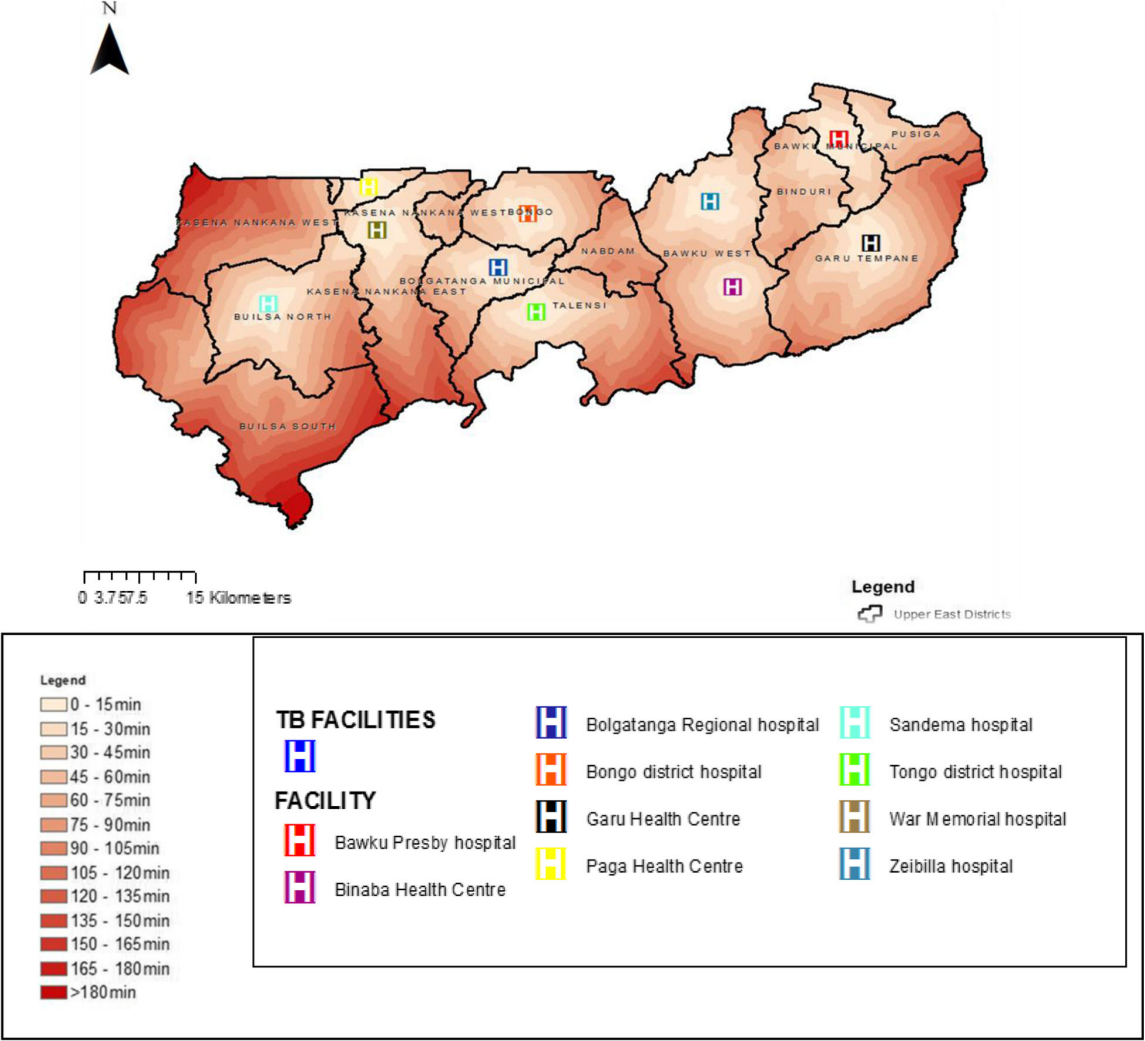
Map showing accessibility to health facilities offering tuberculosis diagnostic services in the Upper East Region

Figure 3 shows 13 modelled surfaces estimating distances values from the population to existing health facilities offering TB testing services in the UER. The maps provide visual prominent indications of the degree of variations in levels of geographical accessibility in relation to distance in the region. Of the 13 districts, 4 (31%) districts namely; Nabdam, Binduri, Builsa South, and Pusiga did not have any facility offering TB test. Suspected TB patients residing in Pusiga district would have to travel to the nearest health facility (Bawku Presbyterian hospital) which is situated in neighboring Bawku municipal for TB testing services. Suspected TB patients living in Binduri district however are bounded by four health facilities providing TB testing services: to the West by Zebilla Hospital (Bawku West), to the South-West by Binaba Health Centre (Bawku West), to the South-East by Garu Health Centre (Garu-Tempane), and to the North-East by Bawku Presbyterian Hospital (Bawku municipal). Nabdam district is also bordered by four districts (Bolgatanga Municipal, Bawku West, Talensi and Bongo) which have health facilities providing TB testing services and suspected TB patients referred from PHC clinics for testing would have to travel to the nearest TB facility. Patients resident in the Northern part of Talensi would be better served (by a short distance) if they opt to visit Zebilla hospital in the Bawku West district as the estimated distance according to the cost distance algorithm would be generally between 20 to 25 km. Patients residing in the central and southern part of Nabdam would have the most difficulty in reaching any health facility providing TB testing services partly due to lack of good roads to allow for faster travel and hence reduced travel distance. The spatial analysis reveals that all TB suspected patients’ resident in the central and southern part of Nabdam district are at least 30 km away from the nearest health facility providing TB testing. The closest referral health facility offering TB testing services to people living in the Builsa South is Sandema hospital which is averagely 30 km from all residential areas in the district.

Table 2 shows the distance and travel time distribution per population in the UER. Cumulatively, the spatial analysis shows that just about 38% of the population residents in the UER have good geographic access (less than 10 km) to a health facility providing TB testing services. Majority (62%) of suspect TB patients referred from PHC clinics for TB testing in the region however will have to travel more than 10 km to access a facility for the service. The mean distance ± SD to the nearest public health facility providing TB testing services in UER was 33.2 km ± 13.5. Whilst the mean travel time ± SD using a motorized tricycle speed of 20 km/h to the nearest facility providing TB testing services in the UER was 99.6 min ± 41.6. The longest mean distance and travel time was recorded in the Builsa South district that is, 53.8 km ± 13.1 and 161.4 min ± 40.2 respectively. Bawku Municipal recorded the shortest distance and travel time to the nearest facility providing TB testing services in the region that is, 21.1 km ± 8.3 and 63.3 min ± 25.7 respectively, as illustrated in Table 3.

**Table 2:** Distribution of distance and travel time per population in the UER

Distance (Km)	Travel time (Minutes)	Population (N = 1,244,983)	Share of population (%)
0 to 5	0–15	286,346	23%
5 to 10	15–30	186,747	15%
10 to 15	30–45	174, 298	14%
15 to 20	45–60	161,848	13%
20 to 25	60–75	136,947	11%
25 to 30	75–90	99,599	8%
30 to 35	90–105	74,699	6%
35 to 40	105–120	37,349	3%
40 to 45	120–135	24,900	2%
45 to 50	135–150	24,900	2%
50 to 55	150–165	12,450	1%
55 to 60	165–180	12,450	1%
60 to 65	> 180	12,450	1%

**Table 3:** Summary of mean (standard deviation) distance and travelling time to the nearest health facility providing TB testing services in each district

District	Mean/average distance (km)	Standard deviation	Mean /average travelling time (min)	Standard deviation
Builsa North	32.5	17.9	97.5	55.7
Kassena Nankana West	39.8	18.7	119.4	57.4
Kassena Nankana Municipal	33.9	18.5	101.7	57.2
Bolgatanga Municipal	25.7	12.9	77.1	40.4
Talensi	41.6	16.1	124.8	48.2
Bongo	28.1	13.5	84.3	41.6
Bawku West	33.2	16	99.6	48.8
Garu-Tempane	37.6	14.1	112.8	43.4
Bawku Municipal	21.1	8.3	63.3	25.7
Builsa South	53.8	13.1	161.4	40.2
Nabdam	32.9	9.9	98.7	31.1
Binduri	24.3	6.8	72.9	20.7
Pusiga	27.2	10	81.6	30.7

### Proposed sites for targeted improvement of TB POC testing services in the UER

**Fig. 4:**
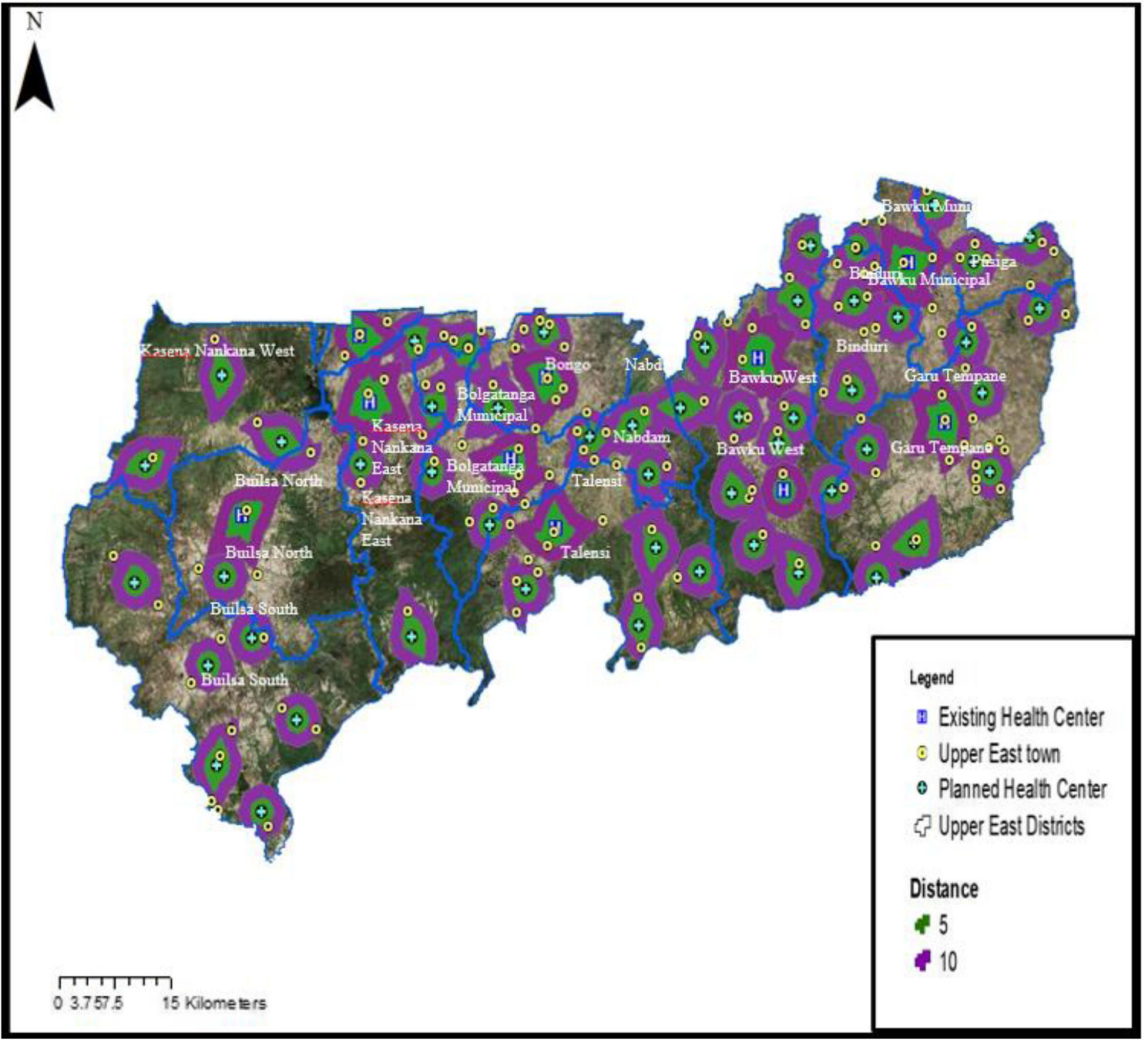
Map showing proposed locations of health facilities for targeted improvement to increase geographic access to TB diagnostic service in the UER

To improve geographic accessibility to TB testing services in the region, this study proposed that there should be at least one health facility providing TB testing services located not more than 10 km from where people live. Base on this, the study employed geographical models to estimate 51 additional healthcare facilities for targeted improvement to meet the unmet TB diagnostic needs of the people resident in the UER. Figure 4 shows a map of the proposed locations of health facilities for targeted improvement to increase geographic access to TB diagnostic service in the UER.

**Fig. 5:**
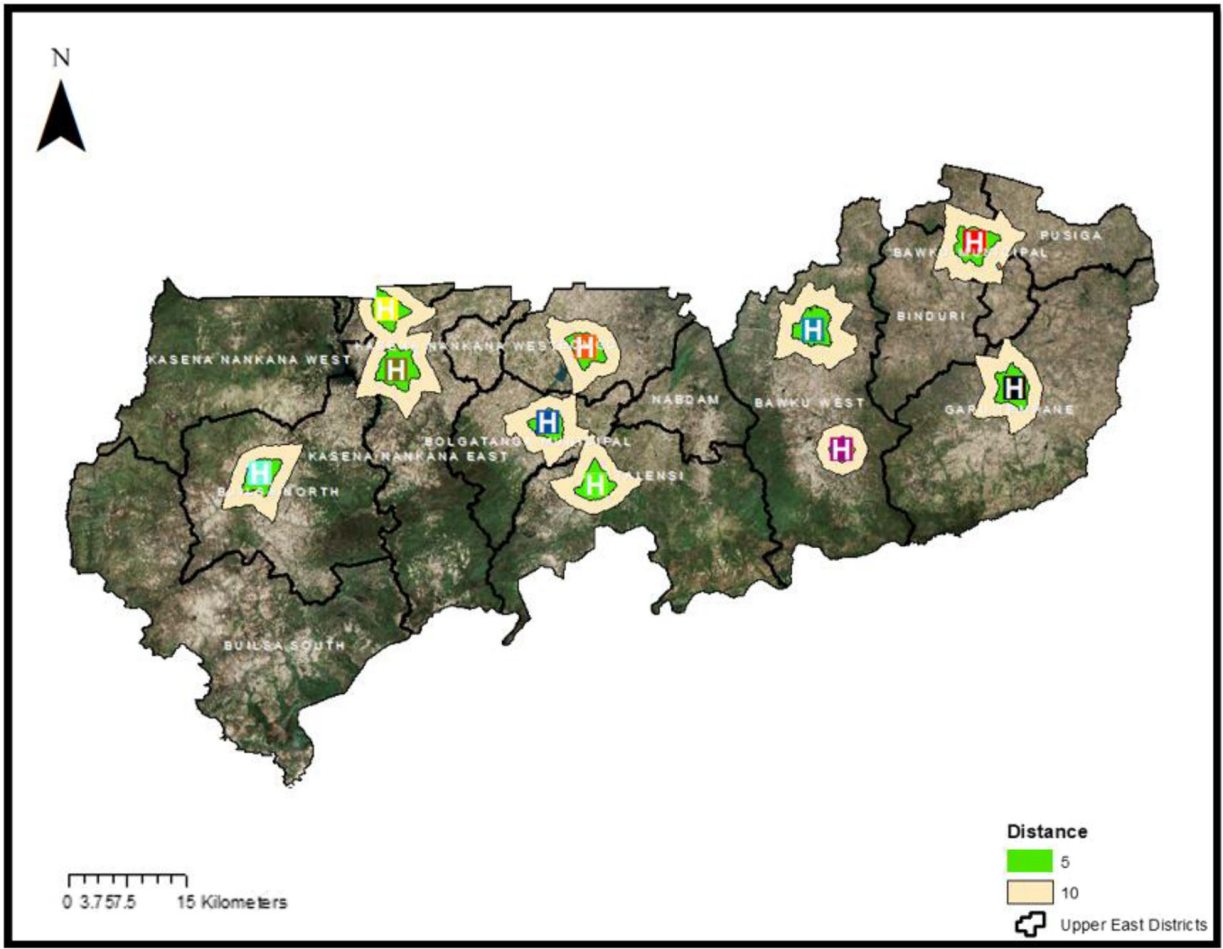
Map showing residential zones and areas within 10 km coverage by existing public health facilities providing TB testing services in the UER

To ensure that all the 51 proposed health facilities would not be in an uninhabited area (example, in the forest), remote sensing (satellite imagery) was first done to map out settlement zones and the areas within 10 km range of the already existing public health facilities providing TB testing services in the UER. An application of the satellite imagery analysis helped to reduce the initially identified 97 health facilities to 51 for targeted improvement to ensure geographical accessibility (less than 10 km) to TB testing services for all residents in the region. The remote sensing classification method of unsupervised classification was used to determine settlement and non-settlement areas (forests). In the supervised classification method, pixels are placed into clusters based on their digital numbers. For example, pixels that capture forest areas are similar and as such, the software classifies all such pixels into a cluster of forest area. The resulting classes were converted from raster to vector data. Using this, a clip function was performed to remove all proposed health facilities that fell in forest areas. Due reduction was achieved after deleting all proposed health facilities falling outside settlements leaving those critical to meeting the unmet diagnostic needs of populations reciting in poor geographical access areas. Figure 5 shows a map of residential zones and areas within 10 km coverage by the existing public health facilities providing TB testing services in the UER.

## Discussion

Geographic access to care is difficult to measure hence the international community most often defined targets based on facility provision per capita [14]. In this study, we assembled unique spatial data on health facilities providing TB testing services, population, and topographic landscape, as well as supporting survey to assess realistic levels of geographic accessibility to public health facilities providing TB testing services at POC in the UER. This study results show majority (62%) of the population travel beyond 10 km to access TB diagnostic testing in the region. It also revealed that TB diagnostic service was available in only ten health facilities in the UER which translates to approximately 125,000 people per facility considering the region’s current total population of 1,244,983. This study further revealed that four districts did not have any health facility providing TB diagnostic services. However, the spatial distribution of the public health facilities providing TB testing services were revealed to be dispersed in the region.

Although geographic accessibility is not the only determinant of access to health care, where service provision is sparse, and the population is predominantly rural, it plays a critical role for healthcare outcomes [14]. Poor geographic accessibility to public health facilities providing TB testing services at POC revealed by this study implies patients in referred from PHC clinics in rural areas have to walk for hours or pay exorbitant transport fares to access TB diagnostic services [15]. Patients often have the tendency to limit or utilize essential health care services closer to them [16]. Majority of rural populations in low and middle income countries are farmers [17]. Therefore, a clients may fail to go for a test or go back for test results when asked to come back the following day or week especially at certain times of the season [18]. Dangisso et al. (2015) study in Ethiopia demonstrated TB case notification rates were higher in areas where people had good access to diagnostic and treatment facilities [15]. Finnie et al. (2011) aimed to identify factors causing delayed diagnosis and treatment for tuberculosis in high TB/HIV burden African countries also demonstrated travel time associated with delays in patients returning for care [19, 20]. Although the spatial distribution of the health facilities providing TB testing services were revealed to be dispersed in the UER, this is deemed desirable to maximize accessibility to TB testing services.

In this study we employed the geospatial methodology to assess the geographical distribution of health facilities providing TB testing services at POC and their proximity to the population. Spatial accessibility measures have been shown to be important policy tool for managing health care provision and reducing health disparities [21, 22]. We used census data from the Ghana statistical service whiles also taking into consideration other geographic data and incorporated remote sensing of aerial data to accurately distribute population within all political boundaries. The application of remote sensing through satellite imagery afforded us the opportunity to observe locations that had settlements as well as enabled us to properly cite proposed health clinics without necessarily having to visit the region. Each population distribution data is unique because it takes into account, the prevailing conditions and spatial nature of each and every region [23]. This study methodology enables us to identify poor geographic access to public health facilities for TB testing services in the UER. Health sector budgetary allocation of countries like Ghana is poor and most often supported by development partners, International organizations, and donors with many competing interests. Therefore, the spatial methodology adopted by this study has strength of informing policy makers to plan and implement TB diagnostic testing services in poor geographic access areas.

Despite the above-mentioned strengths of the study, the following limitations should be noted. Although, we assembled a comprehensive set of data describing the landscape, it is possible we may not have captured all local details such as the prevalence or incidence rate of TB in the UER. We therefore recommend future studies to focus on mapping and determining how many TB cases fall within ‘high’ versus ‘low’ accessible areas in the region. Also, using an assumed motorized tricycle speed of 20 km/hour to calibrate travel time may always not be applicable since other transportation options such as walking, use of a motorbike, bicycle or a car may be utilized by some patients to the health facilities. In addition, traveling time may be affected by seasonal changes, traffic, and other essential factors. Nonetheless, this study provided very useful information to help planning and improve geographical access to TB POC testing particularly for rural populations.

Efforts to reduce TB morbidity, mortality, and catastrophic cost associated with treatment by improving the quality and availability of diagnostic services and treatment in selected health facilities will have limited impact where long distances, poor infrastructure at the PHC level, lack of good roads and transport systems means patients referred from PHC clinics may be unable to access services timely. To improve TB care the WHO recommends TB testing alongside other POC test in resource-limited settings in its maiden list of essential diagnostics [24]. Geographic accessibility is a key aspect of ensuring better health care and treatment for people and clearly, in the case of the UER as demonstrated by this study, a higher proportion of the populace are located at a significantly longer distance away from public health facilities providing TB testing services. Hence, this study proposed an improvement of at least 51 PHC clinics in the UER to enable them provide TB testing services. Improving road networks and making available faster and safer transport systems in rural areas in the region would also greatly improve travel time as well.

## Conclusion

The study findings show poor geographic accessibility to public health facilities providing TB testing services at POC in UER, Ghana. The findings of this study have provided evidence-based information to help with planning and improving TB testing services targeting first rural PHC clinics located in poor geographic access areas in the region. Travelling long distances to access a health facility for TB testing outside one’s location can be a limiting factor for most suspected TB patients and hence, could affect the successes chalked so far toward the end TB strategy in the region. Long distances also may result in late diagnosis and treated as well as worsen the catastrophic cost currently associated with TB treatment in Ghana. To reduce the impact of TB infection, diagnosis, treatment, and related catastrophic cost related in Ghana and other LMICs, the measurement of geographical accessibility is very relevant to provide evidence-based strategies to improve TB care. We will like to recommend a replication of this study in high prevalence regions in Ghana and other TB high prevalence countries. We also recommend TB active case finding in low prevalence regions especially among high risk populations to facilitate achievement of the end TB strategy by 2035.

## Additional file

Additional file 1: Detailed description of model for estimating travel time (DOCX 15 kb)

## Data Availability

Data from this study cannot be shared publicly because it contains sensitive, identifying patient information. All interested researchers/readers/persons who meet the criteria for access to confidential data can access the data set via Dr. Tivani P. Mashamba-Thompson, the project supervisor and the Academic Leader (Research) for the School of Nursing and Public Health via this email address: Mashamba-Thompson@ukzn.ac.za. Data access may also be requested from the University of KwaZulu-Natal Biomedical Research Ethics Committee (BREC) from the following contacts: The Chairperson BIOMEDICAL RESEARCH ETHICS ADMINISTRATION Research Office, Westville Campus, Govan Mbeki Building University of KwaZulu-Natal P/Bag X54001, Durban, 4000 KwaZulu-Natal, South Africa Tel.: + 27 312,604,769 Fax: + 27 312,604,609 Email: BREC@ukzn.ac.za.

## Abbreviations

DEM: Digital elevation model
GOG: Government of Ghana
LMICs: low- and middle-income countries
PHC: Primary Health Care
POC: Point-of-Care
RHD: Regional Health Directorate
SDGs: Sustainable Development Goals
TB: Tuberculosis
UER: Upper East Region
WHO: World Health Organization
