# Supplementary material for: Geographic accessibility to public health facilities providing tuberculosis testing services at point-of-care in the upper east region, Ghana
Source: BMC Public Health. 2019 Jun 10;19:718. doi: 10.1186/s12889-019-7052-2 (PMC6558903; doi:10.1186/s12889-019-7052-2)
Supplement: Supplementary file 1 — Detailed description of model for estimating travel time (DOCX 15 kb) [file 12889_2019_7052_MOESM1_ESM.docx]

**Supplementary file**

**Supplementary file 1:** Detailed description of model for estimating travel time

This was accomplished using a sequence of algorithms with the final output incorporating cost distance tool in ArcGIS 10.4. Cost distance calculates the shortest time to a source based on a cost dataset. To realize this, a cost surface algorithm was designed with these parameters: a grid cell of size 10m was assigned to the spatial features and values were then assigned to the predetermined grids. To ensure that our computing power could adequately handle the processing of the cost distance analysis, there had to be a recalibration of the cell size. Identifying the best cell size for any spatial data relies heavily on the following: the application and analysis to be performed, disk capacity and response time. The cell must be small enough to fully represent the required detail but large enough, so computer storage and analysis can be performed much more efficiently (ESRI, 2008). Several cell sizes bordering around the initial 27

C2 General

cell size (0.6 meters) were applied and we realized it required a much longer time to achieve results and, in some cases, caused a freeze up of all operations. This resulted from the fact that smaller cell sizes required much more processing, and this informed the decision to employ 10 meters. The cost distance run smoothly thereafter, and we were cautious to keep from increasing the cell size any further as we did not want to miss any details.

Proceeding from this, we then had to determine how long it takes to travel per cell. To achieve this, we considered the fact that each cell was set to 10 meters and we therefore had to convert the travel speed using the motorized tricycle which is 20km/h which equates to 20,000 meters. We then had to estimate how many seconds it takes to travel 1 meter and this we achieved by dividing 3,600 seconds by 20,000 meters which equals 0.18 seconds. Our cell size was set at 10 meters so to find out how long it takes to travel each cell we multiplied 0.18 seconds by 10. This meant it took 1.8 seconds to travel via road using the tricycle. The raster calculator was then used in reassigning each cell to 1.8 seconds as the travel time required traveling each cell. Roads would then have a low value (seconds per cell) because traveling on roads is faster than travel via paths and travel over impediments takes a much longer time. This similar method was applied to other land cover areas as this had to be computed and included in the analysis. Since it is hard to travel in areas like water bodies and rocky areas, these were assigned very high travel time per cell in order to ensure an algorithm and results close to realism. To determine travel time per cell for paths, we set the speed at 10km/h. Just as was calculated for travels via road, 10km equates to 10,000 meters and to determine how many seconds it takes to travel 1 meter 3,600 is divided by 10,000 seconds. The resulting figure, 0.36 is then multiplied by the cell size which is 10 and the travel time per cell for paths is 3.6 seconds.

Finally, to realize travel time per cell for land cover features that are considered as impediments and thus require more travel time, we set the speed at 5km/h. This means traveling on these land cover features could be much slower. The speed 5km equates to 5,000 meters and to determine how many seconds it takes to travel 1 meter 3,600 was divided by 5,000 seconds. The resulting figure, 0.72 is then multiplied by the cell size which is 10 and the travel time per cell for impediments is 7.2 seconds. Cell statistics was used to aggregate the varying results obtained from the different thresholds set. 28

C2 General

Since cost distance requires the cost surface dataset and the source, the raster dataset served as the cost surface dataset and health facilities offering TB testing served as the source for calculating the cost distance. The output is a map showing shortest travel time (cell by cell) from any point in the map to a health facility offering TB testing in the region. Algorithms which allowed for carrying out conversion of data from vector to raster, map algebra (cost surface models) and cost distance was developed using Python 2.7. Designing the algorithm helped to avoid repeating the various processes for the varying districts via the ArcMap toolbox. Based on prior information on the most commonly used mode of transportation, the travel speed of a tricycle was pecked at 20 km/h. This served as a guide for determining travel time in the region. Following this, a table was created to depict the average travel time and distance as well as standard deviation for each district. Using the speed of 20km/h all the data obtained from the algorithm was converted to distance.
